# Low parental support in late adolescence predicts obesity in young adulthood; Gender differences in a 12-year cohort of African Americans

**DOI:** 10.1186/s40200-015-0176-8

**Published:** 2015-05-28

**Authors:** Shervin Assari, Cleopatra Howard Caldwell, Marc A. Zimmerman

**Affiliations:** Department of Psychiatry, School of Medicine, University of Michigan, 4250 Plymouth Rd., Ann Arbor, MI 48109-2700 USA; Center for Research on Ethnicity, Culture and Health, School of Public Health, University of Michigan, Ann Arbor, MI USA; Department of Health Behavior and Health Education, School of Public Health, University of Michigan, 2846 SPH I, 1415 Washington Heights, Ann Arbor, MI 48109-2029 USA; Prevention Research Center, School of Public Health, University of Michigan, Ann Arbor, MI USA

**Keywords:** African Americans, Women, Men, Obesity, Parenting, Parental support, Social support

## Abstract

**Background:**

Most studies that have investigated the link between parenting behaviors and risk of obesity among offsprings have mostly used a cross-sectional design, enrolled Caucasian samples, focused on childhood obesity, and covered aspects of parenting behaviors that directly influence energy balance and food intake of the children. Thus, more longitudinal research is needed on how more general aspects of parenting influence obesity in young ethnic minority adults. The current longitudinal study aimed to test if baseline parental support predicts change in body mass index (BMI) of African Americans, and if this prediction varies based on gender of offspring.

**Methods:**

The current study followed 227 young African American adults (109 male and 118 female) for 12 years from year 2000 (mean age 20) to year 2012 (mean age 32). All participants were enrolled from a disadvantaged urban area in the Midwest of the United States. Baseline demographics (age, gender), socio-economics (family structure, and parental employment), psychological symptoms (anxiety and depression), general parental support (maternal support, and paternal support) were measured. BMI was measured at baseline and at follow up. We used gender-specific linear regressions to test the predictive role of baseline paternal and maternal support (year 2000) on change in BMI (from 2000 to 2012).

**Results:**

Regression analysis showed that among female African American young adults, high baseline maternal support was predictive of a lower increase in BMI from 2000 to 2012. The association remained significant while all covariates were in the model. We could not find such an association for male African American young adults.

**Conclusion:**

High maternal support appears to be protective against increases in BMI among African American female young adults. As parental support is a modifiable factor within available evidence-based interventions that enhance parenting, it should be included in obesity prevention programs for African American women. Policies and programs should support African American mothers in disadvantaged neighborhoods to enable them to provide high levels of parental support for their young adult daughters. Future research should test the efficacy of such programs and policies for reducing obesity among African American women.

## Background

Although the cross-sectional link between parents’ behaviors and offsprings’ obesity is known [1–5], it is not known how this association is shaped over time. In addition, although some particular behavior of parents are components of the obesogenic environment for the child [6], it is not clear if more general aspects of parenting also influence risk of obesity of the offspring. Furthermore, it is not clear if this association is independent of confounders such as socio-economic and psychological factors. Finally, less is known about the potential moderating effects of gender for both parent and offspring.

Most studies on the link between parents’ behaviors and offspring obesity have focused on specific parenting behaviors that directly impact energy balance (i.e. food consumption, physical activity, and sedentary behavior) [1–5, 7–12]. However, it is not clear if the effects of parents’ behaviors on offspring obesity are exclusively limited to the particular parenting behaviors that influence feeding, exercise, nutrition, and sedentary life style [13, 14]. General domains of parenting such as support, closeness, and warmth may also protect the child from the effect of threats that may impose risk for obesity among children [15–19]. In addition poor parenting may directly increase risk of obesity for the child, due to the known effect of chronic stress on the development of obesity [20, 21]. We argue that high quality parenting may have a general protective effect, above and beyond parenting behaviors that directly shape life style and energy balance of the child.

Unfortunately very few studies have measured the effect of general parenting on obesity of offsprings [15–19]. We argue that parental support may be one of the ingredients by which high quality parenting may protect the child against obesity. Hypothetically, supportive parenting may protect the child against exposure and susceptibility to adversity [22]. In this view, parenting support may be particularly protective for children who are living in harsh environments and are commonly exposed to chronic stress, which is known to be obesogenic [23–26].

We use a social support framework [27] to guide the current study. Based on this framework, emotional support has a direct protective effect on health outcomes, and also mitigates the negative effect of stressors [27]. Although residents of disadvantaged areas are exposed to high levels of environmental stress, they may perceive their environment as less stressful if they have access to interpersonal support [28]. This is in line with multiple studies suggesting that offsprings who receive higher levels of parental support report lower levels of perceived stress and psychological distress [28, 29].

Only a handful of studies have ever focused on the influence of parental support on obesity among adults [30, 31]. In 2011, Vámosi et al. enrolled adult same-sexed twin pairs discordant for BMI from the Danish Twin Registry to test if general parental support was associated with obesity in adults. Their study showed that recalled maternal antipathy and maternal neglect were associated with adult obesity. However, paternal neglect and antipathy were not related to adult obesity [30]. As psychological distress was associated with both parenting and obesity, the results of the study by Vámosi et al., may have been confounded by the psychological status of the participants.

The current longitudinal study was conducted to test if parental support at age 20 was predictive of change in BMI from age 20 to 32 among male and female African American young adults, while the effect of socio-economic status and psychological distress were controlled. We hypothesized that the association between parental support (i.e., mother and father) and change in BMI over time would depend on the gender of the offspring.

## Materials and methods

### Design

Our data came from the Flint Adolescent Study (FAS), a long-term cohort study in Flint Michigan. The study protocol was approved by the University of Michigan Institutional Review Board. All participants signed consent or assent forms before each interview.

Data for the current study came from years 2000 and 2012 when participants were an average age of 20 and 32, respectively. The original study enrolled participants from the ninth grade (age 15) to their transition into adulthood; however, the first measure of body mass index was measured when participants were 21 years old on average.

### Participants

Participants were sampled from four local public high schools. The study enrolled students in the fall semester of ninth grade if they had a grade point average (GPA) of 3.0 or lower in eighth-grade and if they did not have a diagnosis of a developmental disability or emotional impairment. Although the original study included African Americans (80 %) and Caucasians (17 %), the current study only included African American men and women to focus on an understudied population in BMI research.

### Procedure

Data were collected using structured face-to-face interviews conducted either at school or at alternative community locations. This study followed students who remained in school, as well as those who dropped out of school. Each interview lasted about 60 min on average.

#### Covariates

Baseline age, family structure (i.e., intact vs. not intact family) and family socio-economic status (number of parents who were employed) were used as control variables.

#### Maternal support

Maternal support was measured using a five item modified version of the parental support scale [32]. All items were modified to include “mother” instead of “parent”. Using a 5-point Likert scale (1: not true, 5: very true), respondents indicated the extent to which they believed they receive support from their mothers. Areas of support included emotional support, problem solving support, and moral support. Sample items include “I rely on my mother for emotional support” and “My mother is good at helping me solve problems.”

#### Paternal support

Parental support was measured using a five item modified version of the parental support scale [32]. All questions used the term “father” instead of “parent”. Items asked respondents the extent to which they believe they receive support from their fathers. All items used a 5-point Likert scale (1: not true, 5: very true). Areas of support included emotional support, problem solving support, and moral support. Sample items include “I rely on my father for emotional support” and “My father is good at helping me solve problems.” (Cronbach’s alpha = .92 at baseline).

#### Body mass index

Body mass index was measured using self-reported weight and height. BMI calculated based on self-reported weight and height is valid and reliable [33, 34]. Several other studies have relied on self-reported weights and heights [35–38]. As weight and height where measured in pounds and inches, respectively, we used the following formula to calculate BMI: BMI = [(weight in pounds) ÷ (height in inches × height in inches)] × 703.

#### Symptoms of anxiety

Symptoms of anxiety were measured by the Brief Symptom Inventory [39]. Six items assessed the frequency of feeling uncomfortable due to symptoms of anxiety during the past week. Response options were on a Likert scale that ranged from 1 (not at all uncomfortable) to 5 (extremely uncomfortable). Items were averaged to form a scale. This scale has been shown to have high internal consistency and test–retest reliability [40, 41]. (Cronbach’s alpha = .78 at baseline).

#### Symptoms of depression

Depressive symptoms were measured by six items from the Brief Symptom Inventory [39]. These items assess the frequency of feeling uncomfortable during the past seven days due to symptoms of depression such as feeling hopeless about the future, and having no interest in things. Response options on the Likert scale ranged from 1 (not at all uncomfortable) to 5 (extremely uncomfortable). These six items were averaged to form the final scale. This scale has high internal consistency and test–retest reliability and is valid to use with adolescents [40, 41]. (Cronbach’s alpha =.79 at baseline).

#### Data analysis

We used SPSS 20 (IBM Corp) for data analysis. Descriptive statistics for age, parental support, and BMI were provided using mean and standard deviations (SD). Pearson’s correlation was used to measure bivariate associations between the study variables. We fitted multiple linear regressions to test if baseline maternal support (year 2000) predicted change in BMI level from baseline to the end of follow up (year 2012). We did not include paternal support in our multivariable analysis because 113 participants did not know or were not in contact with their fathers. We also ran similar models specific to each young adult’s gender to test if these associations varied by gender. In all models, age, number of employed parents, and family structure at baseline were included as controls. P-values less than 0.05 were considered significant. Beta (correlation coefficient) and a 95 % Confidence Interval (CI) were reported.

## Results

### Descriptive statistics

Participants had a mean age of 20 years at baseline. Mean age was 32 at follow up. Baseline symptoms of anxiety, depression, BMI, and change in BMI varied based on gender. All measures listed were higher among females (see Table 1).

**Table 1:** Descriptive statistics among all, male and females

	Male		Female		All	
	Min-Max	Mean ± SD	Min-Max	Mean ± SD	Min-Max	Mean ± SD
Baseline Age	18.87-21.85	19.94 ± .66	18.89-21.88	19.79 ± .62	18.87-21.88	19.86 ± .65
Baseline Intact family	0.00-1.00	.29 ± .45	0.00-1.00	.21 ± .41	0.00-1.00	.25 ± .43
Baseline Parental employment	0.00-1.00	.50 ± .50	0.00-1.00	.45 ± .50	0.00-1.00	.47 ± .50
Baseline Maternal Support	1.00-5.00	4.11 ± .94	1.00-5.00	3.96 ± 1.07	1.00-5.00	4.03 ± 1.02
Baseline Paternal Support	1.00-5.00	3.36 ± 1.15	1.00-5.00	2.98 ± 1.24	1.00-5.00	3.14 ± 1.22
Baseline Symptoms of Anxiety	1.00-3.67	1.51 ± .55	1.00-5.00	1.63 ± .65	1.00-5.00	1.58 ± .612
Baseline Symptoms of Depression	1.00-4.67	1.64 ± .64	1.00-5.00	1.83 ± .76	1.00-5.00	1.75 ± .71
Baseline BMI	15.81-56.48	25.50 ± 5.43	16.72-54.85	27.14 ± 7.13	15.81-56.48	26.42 ± 6.49
Follow up BMI	17.93-61.00	29.40 ± 7.37	17.12-68.63	32.29 ± 9.42	17.12-68.63	31.09 ± 8.73
BMI Gain	−5.54-23.30	3.94 ± 4.65	−11.15-25.03	4.19 ± 5.55	−11.15-25.03	4.10 ± 5.21

### Bivariate analysis

In bivariate analyses, BMI change was not associated with age, parental support, or symptoms of anxiety or depression among males. BMI change was not associated with age, paternal support, or symptoms of anxiety and depression among females. BMI change was, however, associated with maternal support among females (see Table 2).

**Table 2:** Correlation matrix of the variables among males and females

	1	2	3	4	5	6	7	8	9	10
1 Baseline Age	1	-.099	-.100	-.123	-.055	.078	-.007	.066	-.062	-.119
2 Baseline Intact family	-.123^*^	1	.146^**^	.192^**^	.169^*^	-.025	-.010	.118	.084	-.080
3 Baseline Parental employment	-.098	.128^*^	1	.045	-.004	-.129	-.115	-.055	.021	.080
4 Baseline Maternal Support	.015	.044	.016	1	.361^**^	-.236^**^	-.290^**^	-.001	.097	.036
5 Baseline Paternal Support	-.038	.163^*^	.091	.265^**^	1	-.052	-.142	-.033	.077	.188
6 Baseline Anxiety	.043	-.021	-.080	-.168^**^	-.138^*^	1	.689^**^	-.019	-.221^*^	-.148
7 Baseline Depression	.060	-.073	-.087	-.294^**^	-.279^**^	.737^**^	1	.059	.031	.008
8 Baseline BMI	.041	.024	-.113	.021	-.014	.019	-.032	1	.795^**^	.177
9 Follow up BMI	-.002	-.014	-.194^*^	-.075	.062	-.002	-.021	.814^**^	1	.737^**^
10 BMI Gain	-.127	-.046	-.160	-.218^*^	-.024	.055	.094	.059	.628^**^	1

Results above and below diagonal is for males and females, respectively

*p < 0.05

**p < 0.01

### Regression analysis

Among females, higher maternal support at baseline predicted lower BMI at follow up. The association remained significant while all covariates were controlled. We could not find such an association among males (see Table 3).

**Table 3:** Summary of regression among the pooled sample, males and females

			95.0 % Confidence interval for B		
	Standardized coefficients	Std. Error	Lower bound	Upper bound	Sig.
All					
Baseline Age	-.085	.656	−2.549	.038	.057
Gender	-.009	.795	−1.741	1.396	.828
Baseline Intact family	-.057	.857	−2.770	.612	.210
Baseline Parental employment	-.026	.600	−1.538	.831	.556
Baseline Maternal Support^a^	-.070	.349	−1.255	.122	.106
Baseline BMI	.813	.061	1.016	1.255	.000
Men					
Baseline Age	-.100	.931	−3.062	.651	.200
Baseline Intact family	-.126	1.224	−4.392	.488	.115
Baseline Parental employment	.028	.860	−1.394	2.035	.710
Baseline Maternal Support^a^	.049	.548	-.734	1.449	.516
Baseline BMI	.785	.108	.916	1.346	.000
Women					
Baseline Age	-.068	.913	−2.921	.700	.227
Baseline Intact family	-.030	1.192	−2.988	1.739	.601
Baseline Parental employment	-.055	.833	−2.468	.836	.330
Baseline Maternal Support^a^	-.122	.458	−1.943	-.126	.026
Baseline BMI	.809	.076	.972	1.272	.000

Although bivariate results are reported for paternal support, paternal support is not used in the multivariate analyses due to missing data

## Discussion

The current study suggests that among female African American adults, low maternal support at age 20 predicts higher BMI increase in the 3^rd^ decade of life. However, a similar association could not be found for males. The association remains significant while socio-economic status, family structure, anxiety, and depression were controlled.

For at least five reasons, our study is a unique contribution to the literature. First, most of the literature on the association between parenting and offspring obesity is focused on infants and young children, while our study focuses on young adults transitioning into midlife. Second, most previous studies have had an emphasis on parenting styles rather than parental support. Third, most of the literature is composed of cross-sectional studies. Forth, few studies have controlled for psychological health of the offspring. Fifth, less is known about potential gender differences in this regard.

In support of our findings, Barnett et al. [16] followed a nationally representative sample of 37,577 Canadian children and measured parenting styles and children’s body mass index (BMI). The study found that compared to authoritarian parenting (setting strict limits, low communication and low affection), authoritative parenting (affectionate, reasonable discussions and set healthy boundaries) may be protective against obesity. The study suggested that authoritative parenting may be associated with a 30 % lower chance of being obese among children 2 to 5 years old and a 37 % lower chance of among children 6 to 11 years. The study suggested that a balance between affection and setting limits may be protective against obesity [16].

The literature on parenting styles with children [42–44] has indicated that authoritative parenting may be associated with low risk for child obesity [45, 46]. It has been suggested that this effect may be due higher levels of fruit consumption [10] and physical activity [9] among those who have authoritative parents.

A study of Mexican American families showed that indulgent mothers were significantly more likely than authoritative, or authoritarian, mothers to have children who became overweight 3 years later [15]. This finding was in contrast with findings of another study that found that authoritarian parenting style was the best predictor of weight gain in their predominantly middle class, European-American sample [45].

Barlow suggested that interventions should encourage authoritative parenting style in support of increased physical activity and reduced sedentary behavior (authoritative parents provide both tangible and motivational support for children, and are also demanding and responsive). He argued that interventions should also discourage a restrictive parenting style (restrictive parenting involves heavy monitoring and controlling of a child’s behavior) [47].

Our findings extend the results of numerous studies confirming a relation between parenting behaviors and positive child outcomes [44]. Studies differ, however, on the parenting styles associated with the highest obesity risk. A study found that children of authoritarian parents (low nurturance and high control) were at the greatest risk [45]. In contrast, another study found that children of indulgent (high nurturance and low control) and uninvolved (low nurturance and low control) parents showed the greatest risk [46]. Because parents’ attitudes toward child rearing are influenced by cultural norms and contextual factors, the effects of different parenting styles often vary across ethnic groups [48, 49]. Few studies have examined parenting and obesity among minority groups [15, 50].

The current study is different from mainstream literature in several ways. Most previous studies in this area have specifically focused on the influence of parents on the food consumption, physical activity, and sedentary behavior of young offspring [9, 10]. For instance, a study found that for Mexican American mothers, high parental control in feeding was inversely correlated with child BMI [51], and in two separate studies, Hughes and colleagues found that children of mothers with indulgent feeding styles had the highest BMIs [12, 19].

Parenting behaviors that are closely associated with energy intake control have been shown to be associated with obesity, however, less is known about parenting more broadly defined, including warmth, support, and closeness. There are few studies testing the effect of general parenting on obesity [15, 16], and fewer studies have tested the association while other possible confounders are controlled. In brief, studies have suggested that a stressful emotional or neglectful childhood is related to adiposity in childhood and also adulthood, taking into account childhood factors, such as socioeconomic position [31].

Other than feeding, exercise, nutrition, and sedentary life style that are directly associated with obesity, there may be other domains of parenting (such as closeness and support) that may protect the child from the effect of threats that may impose risk of obesity [15–19]. In this view, abusive relation or harsh parenting may act as a stressor and directly increase risk of obesity [21]. The effect of abusive parenting on obesity has been also shown [21, 52]. Thus, the protective effect of the parent may not be because of their influence on life style factors that directly influence energy balance, but be because of the protective parental support that has a main or buffering effect against negative effects of stressors [27].

In a prospective study of 9310 members of the 1958 British birth cohort who were interviewed at 45 years of age, risk of obesity increased between 20 % and 50 % for adversities such as physical abuse, verbal abuse, witnessed abuse, humiliation, neglect, strict upbringing, physical punishment, conflict or tension, low parental aspirations or interest in education, hardly takes outings with parents, and father hardly reads to child. For most associations, socioeconomic factors partially explained the link between childhood adversity and adulthood obesity [31].

A study by Olvera and Power [15] examined in a low-income, Mexican American sample, the relations between parenting style and the development of child weight status 3 years later. The study consisted of 80 Mexican American mother–child dyads that participated in a 4-year study [53] and showed links between parenting styles and children’s weight status in a 4-year study of low-income, Mexican American families. The results showed that indulgent mothers were significantly more likely than authoritative or authoritarian mothers to have children who became overweight 3 years later [15]. Their findings were in contrast with the findings of another study that found that authoritarian parenting style was the best predictor of weight gain [45]. Although the first study used a minority sample, the second study enrolled a predominantly middle class, European-American sample.

Low socioeconomic status has always been linked to parenting [54, 55] and obesity [56, 57], thus socioeconomic status may confound the effect of parenting behaviors on obesity of the offspring. It is, not known if the parenting of African Americans that is described as punitive, less consistent, authoritarian, and often including physical discipline [58] is due to their low socio-economics or not. There are studies suggesting that the differences in parenting by racial groups may be a response to the contextual necessities and opportunities associated with lower socio-economic status [59, 60]. From studies that have disentangled the effects of race from socio-economic status, some suggest it is socio-economics and education, but not race – per se-, that shape the parenting behaviors of low income African American mothers [61]. Levels of warmth, conflict, and permissiveness that children experience is directly affected by family structure [62].

The study findings are supported by a life course perspective [63–65] which suggests that early exposures in childhood and adolescence influence risk of adult chronic conditions. Adolescence and young adulthood should be considered a sensitive developmental stage, and exposure at this period may shape life course trajectories later in life. In this study, we showed that parental support at age 20 will have long-term health consequences for obesity later in life [65]. These findings are particularly important because obesity of young adults have important implications for developing chronic conditions later in life [63].

The result of this study was specific to African Americans and should be tested for replication among other social groups. Race and culture shape parenting style and behaviors among parents [66, 67] and also vulnerability of the offspring to the parenting behaviors [17, 68–71]. Emotional expressivity is influenced by environmental context. Issues such as violent neighborhoods may explain the results of studies that describe the parenting behaviors of African Americans as less warm and less sensitive compared to Caucasians [72]. The parenting style used by African American mothers has been described as positive control where mothers tend to set clear and firm rules and to establish themselves as authority figures [73] in efforts to protect their children. Latino parents have also been characterized as showing high levels of parental control [74–77].

In our study, the negative association between parental support and the development of obesity was limited to female children. Thus, gender of the child changes the vulnerability to the effect of parenting support on obesity [78]. Parents may more frequently employ authoritarian parenting style with boys than with girls [79]. Among African Americans, studies of ethnically diverse families have suggested that African American mothers may use higher levels of negativity and detachment with boys [80]. One study has suggested that maternal but not paternal antipathy and neglect were associated with adult obesity [30].

Olvera and Power provided three possible explanations for why children of low-income minority indulgent mothers may be at highest risk for the development of obesity. First, indulgent mothers may show low levels of control in the feeding context as well; therefore, children may be more frequently exposed to obesogenic foods. Second, children of indulgent parenting may not have the same level of development of self-regulation in eating and exercise [15, 79]. Finally, indulgent mothers may cater more to their children’s unhealthy food preferences or may less frequently encourage children to partake in physical activity [15].

The current findings may have clinical and public health implications for practice. Policy makers may consider policies that support positive parenting among African American women who are living in disadvantaged areas. Family practitioners who work with African American families (particularly single mother families) may design interventions that target enhancement of parenting and parental support as a possible strategy for prevention of obesity among African American women. Family based interventions that enhance parenting may also have implications for obesity prevention among African American women. Interventions that enhance positive parenting and improve parental support are available [81–83]. Based on our findings, positive parenting should be also enhanced. The effect of public health programs and policies that enhance parental support among women may have different effects on the obesity of African American female adults than male adults.

Maternal support is a modifiable factor that can be enhanced by interventions. There is well-established literature on programs that effectively enhance parenting [84]. Thus, maternal parenting should be considered as a target for interventions that wish to prevent or reduce obesity among African American women. As there are available interventions that enhance parental support, policy makers, public health programmers and family practitioners may have multiple options to boost parenting support among African American women. Policies are needed to be implemented to enhance parental support among African Americans who are living in disadvantaged neighborhoods.

Future research should replicate our findings in large, diverse samples. Research should also examine the specific mechanisms that account for the relations between parental support and less obesity among minorities [15]. Future research should also explore possible reasons behind gender differences in these areas.

The current findings may help with the prevention of obesity among African American women. In 2011, compared to Caucasians, African Americans were 50 % more likely to be obese, while, the additional risk was 80 % for African American women. This indicates that about four out of five African American women are overweight or obese [20]. In a dose dependent fashion, obesity increases the risk of several cardiovascular outcomes [85]. A paper published in JAMA in 2013 showed that time lived with obesity may also increase the risk of death from cardiovascular causes, independent of BMI [86]. Obesity is known to increase the risk of hypertension [87], diabetes [88], metabolic syndrome [89], cardiovascular disease (CVD) [90], and stroke [91].

The current study is not free of limitations. BMI was based on self-reported weight and height. Our study only used based line measures of family structure, socio-economic status, and parenting quality, however all these constructs are subject to change. The study also did not include other measures of family socio-economic status such as family income. Despite these limitations, the longitudinal design, long term follow up, a focus on more general aspects of parenting and obesity of young adults, and enrolment of ethnic minorities make the current study a unique contribution to the field.

Our findings are particularly important due to the developmental stage of our participants. Young adults who transition into midlife deal with developmental tasks which are unique to their stage of development. Although they are not as dependent on their mothers for behaviors relevant for obesity like the younger children, maternal support still contributes to their risk of obesity. Thus the effect of parent on obesity of the offspring is beyond childhood and extends to young adults.

To conclude, higher maternal support at age 20 is predictive of less increase in BMI over the 3rd decade of life among African American females but not males. Policies and practices that may enhance maternal support may contribute to prevention of obesity among African American women. Further research should investigate why the effect is not evident for male African American young adults.
